# A Rare Case of Adrenocorticotropic Hormone (ACTH)-Secreting Pheochromocytoma Presenting With Severe Cushing Syndrome (CS) and Dual Hormonal Secretion

**DOI:** 10.7759/cureus.97772

**Published:** 2025-11-25

**Authors:** Yi Yi Aung, Nicholas Hutton, Sawsan Hamdan, Robert Hadden, Tarig Babiker

**Affiliations:** 1 Diabetes and Endocrinology, University Hospitals Plymouth NHS Trust, Plymouth, GBR; 2 Emergency Medicine, University Hospitals Plymouth NHS Trust, Plymouth, GBR; 3 Cellular and Anatomical Pathology, University Hospitals Plymouth NHS Trust, Plymouth, GBR

**Keywords:** acth secreting tumor, dual hormone secretion, ectopic cushing's syndrome, hypokalemia, phaeochromocytoma

## Abstract

We present a rare case of a 58-year-old woman diagnosed with an adrenocorticotropic hormone (ACTH)-secreting pheochromocytoma, manifesting as severe Cushing syndrome (CS) with dual-hormonal secretion. She had a three-week history of upper-body tremors, hyperglycemia, hypokalemia, and new-onset, treatment-resistant hypertension. Biochemical investigations revealed markedly elevated cortisol, ACTH, and catecholamine levels. Imaging identified a 3.4 cm enhancing left adrenal mass, and further functional imaging and hormonal assays confirmed the diagnosis. She underwent laparoscopic adrenalectomy following medical stabilization with alpha-blockade and metyrapone. Histopathology confirmed a low-risk pheochromocytoma with positive ACTH immunostaining. Postoperatively, the patient had complete resolution of hypercortisolism, normalization of metabolic parameters, and significant clinical improvement. This case highlights the importance of recognizing dual-hormonal secretion in adrenal tumors, as early detection allows safer preoperative optimization and reduces perioperative risk. It also emphasizes maintaining a high index of suspicion for ectopic ACTH production in rapidly progressing CS. Early diagnosis and appropriate preoperative management are critical to reducing morbidity and mortality.

## Introduction

Pheochromocytomas are rare neuroendocrine tumors arising from chromaffin cells that typically secrete catecholamines. However, ectopic hormone secretion, such as adrenocorticotropic hormone (ACTH), is extremely rare and may lead to paraneoplastic Cushing syndrome (CS) [1]. Recognizing this presentation is critical, as hypercortisolism can exacerbate catecholamine effects and increase perioperative risk. This case contributes to the limited literature on ACTH-secreting pheochromocytomas. 

## Case presentation

A 58-year-old woman presented to her general practitioner with a three-week history of upper-body shaking and unsteadiness. Her medical history includes non-alcoholic fatty liver disease, hypertension, pre-diabetes, depression, and psychosis. Regular medications included mirtazapine 30 mg at night, once daily vitamin D 800 units, and ramipril 10 mg. On examination, she had a BMI of 22.15, a blood pressure of 198/113 mmHg, facial plethora, hirsutism, and rounded facial features, but no striae or dorsocervical fat pad. She appeared anxious and distressed. 

Investigations 

Admission blood tests revealed blood glucose of 23.1 mmol/L, ketones 1.8 mmol/L, potassium 2.8 mmol/L, and glycosylated hemoglobin (HbA1c) 77 mmol/mol (previously 44 mmol/mol two months prior) (Table 1). A random cortisol level was 2335 nmol/L. These findings prompted hospital admission. Endocrine workup ruled out primary aldosteronism. Other hormonal assays showed elevated testosterone and mildly raised PTH (Table 2). During the course of the investigation, our patient’s thyroid function test remained within range. One retrospective cohort study reported that biochemical central hypothyroidism (CH) resolved in 53% of cases six to 12 months after a cure [2]. 

**Table 1:** Investigation at presentation VBG: venous blood gas; HbA1c: glycosylated hemoglobin

Investigation	Result	Reference range	Interpretation
Haemoglobin (g/L)	165	120-160	Normal
White blood cells (x10^9^/L)	12.4	4-11	Mildly elevated
Platelet (10^9^/L)	157	150-400	Normal
Mean corpuscular volume (fL)	85.5	82-100	Normal
Random blood glucose (mmol/L)	23.1	3.9-5.8	Elevated
HbA1c (mmol/mol)	77	<42	Marked deterioration (previous 44)
Ketones (mmol/L)	1.8	<0.6	Mildly elevated
Sodium (mmol/L)	142	133-145	Normal
Potassium (mmol/L)	2.8	3.5-5.0	Persistent hypokalemia
Urea (mmol/L)	9.7	2.5-7.8	Normal
Creatinine (µmol/L)	69	49-90	Normal
Glomerular filtration rate (mL/min/1.73m^2^)	84	>60	Normal
pH (VBG)	7.54	7.35-7.45	Metabolic alkalosis
Bicarbonate (mmol/L)	35.4	22-26	Metabolic alkalosis

**Table 2:** Endocrine Profile ACTH: adrenocorticotropic hormone; DHEAS: dehydroepiandrosterone sulfate

Investigation	Result	Reference range	Interpretation
Random cortisol (nmol/L)	2335	140-690	Significantly elevated
9 am cortisol (nmol/L)	2976	140-690	Confirmatory for hypercortisolism
24-hour urine cortisol (nmol/24hr)	10170	0-165	Markedly elevated
ACTH (ng/L)	483	0-46	Suggest ectopic secretion
DHEAS (µmol/L)	3.23	0.0-3.7	Borderline high
Aldosterone (pmol/L)	145	23.2-388.6	Normal
Renin (mU/L)	18.6	4.0-48.9	Normal
Aldosterone: renin ratio	7.8	<91	Excludes primary aldosteronism
Prolactin (mIU/L)	187	109-557	Normal
Thyroid-stimulating hormone (mIU/L)	0.65	0.35-4.94	Normal
Plasma free metadrenaline (pmol/L)	2079	0-510	Elevated
Plasma free normetadrenaline (pmol/L)	1870	0-1180	Elevated
Plasma free 3-methoxytryramine (pmol/L)	235	0-180	Elevated
24-hour urine noradrenaline (µmol/24hr)	7.28	0.0-2.0	Elevated
24-hour urine normetadrenaline (µmol/24hr)	4.74	0-4.3	Mildly elevated
24-hour urine 3-methoxytryramine	2.52	0-2.6	Borderline elevated
Testosterone (nmol/L)	2.4	0.5-1.2	Elevated
Growth hormone (ug/L)	0.07	Variable	Suppressed
Follicular stimulating hormone (IU/L)	0.5	Post-menopause >25	Suppressed
Luteinizing hormone (IU/L)	<0.5	Post-menopause >15	Suppressed
Parathyroid hormone (pmol/L)	9.6	1.6-7.2	Elevated
Total 25-OH vitamin D (nmol/L)	27	50-300	Deficient

**Figure 1:**
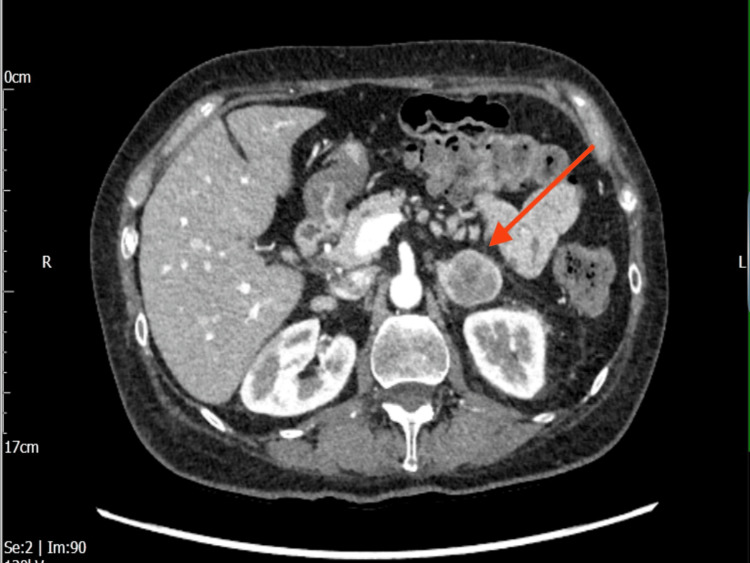
CT biphasic abdomen and pelvis with contrast demonstrating a large, solid, heterogeneous left adrenal nodule measuring 3.4 cm × 2.8 cm (red arrow) CT: computed tomography

**Figure 2:**
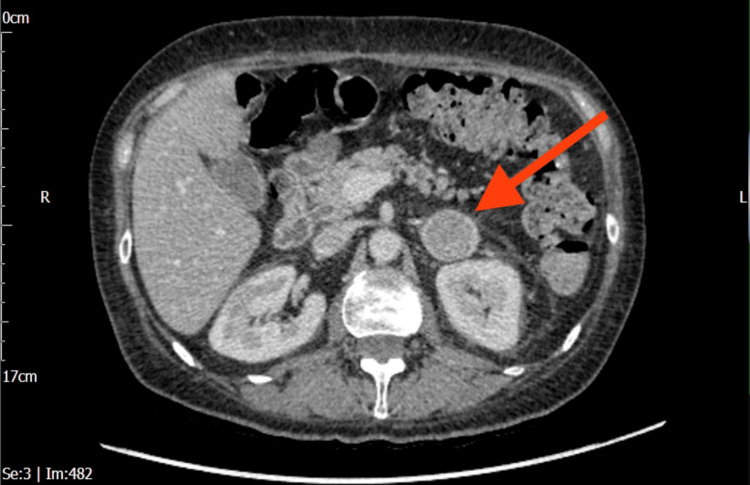
Triple-phase CT of the abdomen demonstrating a left adrenal mass (red arrow). Note the preserved volume of the contralateral adrenal gland CT: computed tomography

**Figure 3:**
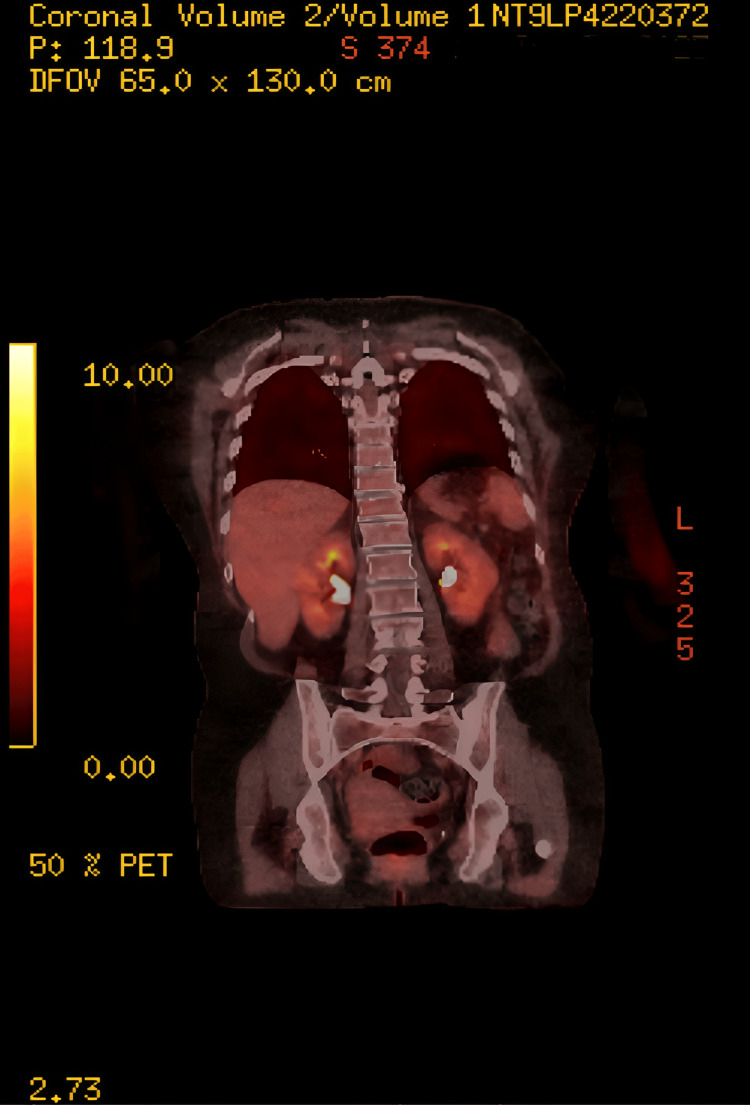
Nuclear medicine whole-body FDG PET/CT revealed a highly avid left adrenal mass. Note the diffuse uptake in the contralateral gland, likely representing an adrenal reaction to hormonal hypersecretion from the nodule FDG PET: fluorodeoxyglucose positron emission tomography; CT: computed tomography

**Figure 4:**
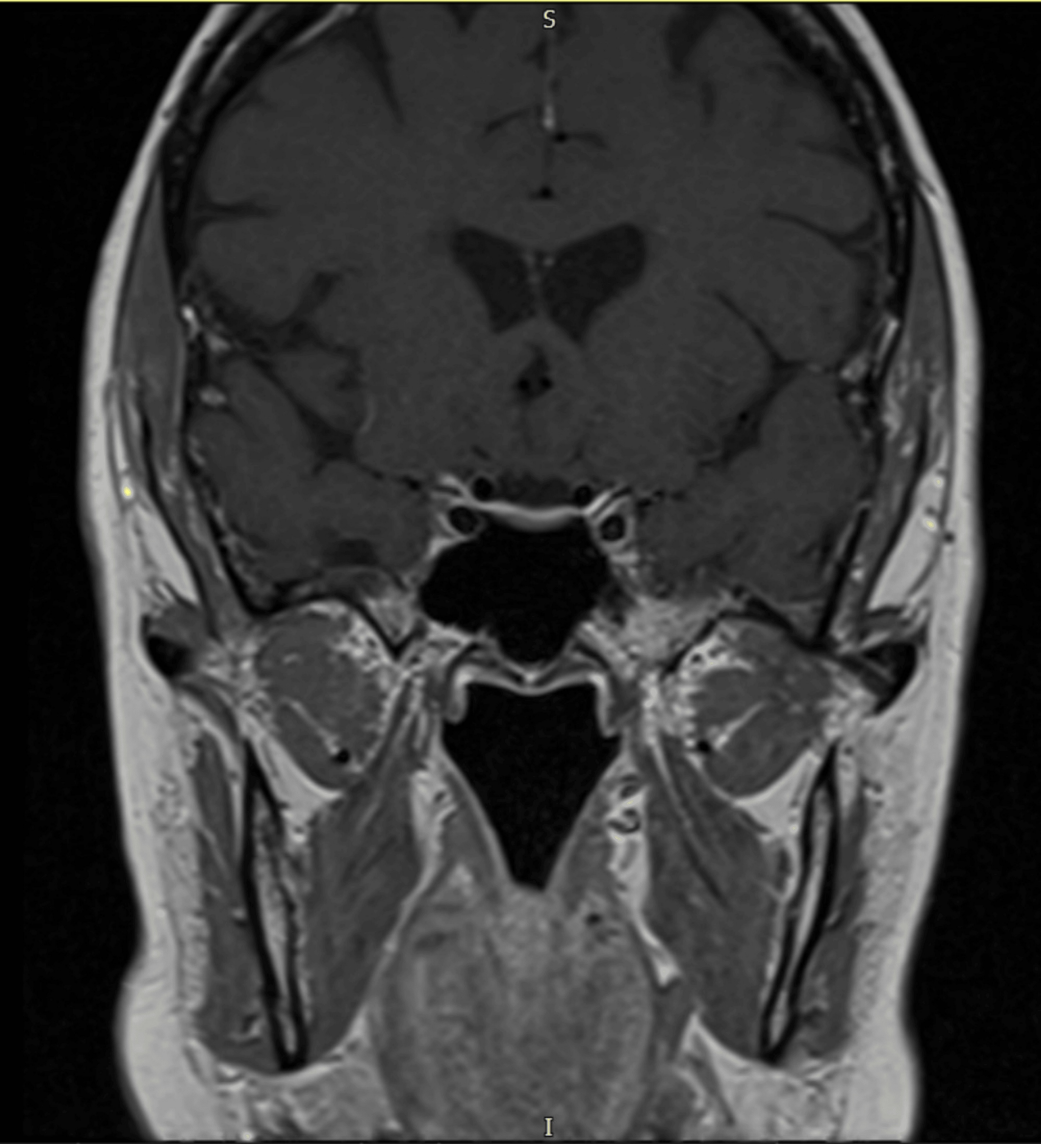
Coronal view of the pituitary MRI showing normal appearance MRI: magnetic resonance imaging

Triple-phase computed tomography (CT) of the abdomen and pelvis demonstrated a left solid, heterogeneous adrenal nodule measuring 3.4 cm × 2.8 cm, which was indeterminate in appearance (Figures 1, 2). A subsequent fluorodeoxyglucose positron emission tomography/computed tomography (FDG-PET/CT) showed a highly fluorodeoxyglucose (FDG)-avid 3 cm left adrenal mass, raising suspicion for either a pheochromocytoma or an adrenocortical carcinoma, with no significant pathological FDG uptake elsewhere (Figure 3). Biochemical assessment revealed markedly elevated serum and urine metanephrines, supporting the diagnosis of pheochromocytoma. A magnetic resonance imaging (MRI) of the pituitary was performed to investigate the source of the elevated ACTH level and demonstrated a normal pituitary gland and fossa (Figure 4).

Treatment 

The patient was diagnosed with an ACTH-secreting pheochromocytoma and started on preoperative alpha blockade [3,4]. Our patient required doxazosin, titrated up to 8 mg daily, and spironolactone 50 mg daily to manage the hypertension. She required a low-dose beta blocker for tachycardia. Preoperatively, she was treated with metyrapone to block cortisol production and keep it within a range of 400-700 nmol/L. She underwent laparoscopic left adrenalectomy during the same admission. In addition to an increased risk of a hypercoagulable state due to acute hypercortisolemia, she had a substantial risk of renal artery thrombus due to CT findings of a thickened, abnormal right renal artery, for which she was started on a treatment-dose oral anticoagulant (rivaroxaban) for three months [5]. She was managed post-operatively in the ITU.

Outcome and follow-up 

Post-operatively, her symptoms resolved, with less anxiety and improved biochemical tests. When assessed two months post-surgery, her blood pressure and HbA1C level had improved significantly, and she was no longer on any medications for hypertension or diabetes. Her catecholamines and metadrenalines had normalized (Table 3). Her anterior pituitary hormone profile recovered to the normal range. However, her short Synacthen test showed suboptimal cortisol response (peak 195 nmol/L), and she remained on replacement hydrocortisone. 

**Table 3:** Hormone profile after adrenalectomy ACTH: adenocorticotropic hormone

Investigations	Result	Reference range
Serum cortisol(nmol/L)	146	140-690
ACTH (ng/L)	29	0-46
Plasma free metadrenaline (pmol/L)	<70	0-510
Plasma free normetadrenaline (pmol/L)	192	0-1180
Plasma free 3-methoxytyramine (pmol/L)	<50	0-180
Thyroid-stimulating hormone (mIU/L)	3.0	0.35-4.94
Free thyroxine (pmol/L)	13.6	12.0-22.0
Follicle-stimulating hormone (IU/L)	93.8	Post-menopause >15
Luteinizing hormone (IU/l)	21.2	Post-menopause >15
Intrinsic growth factor-1 (mol/L)	22.2	5.9-27.5
Prolactin (miU/L)	454	109-557

**Figure 5:**
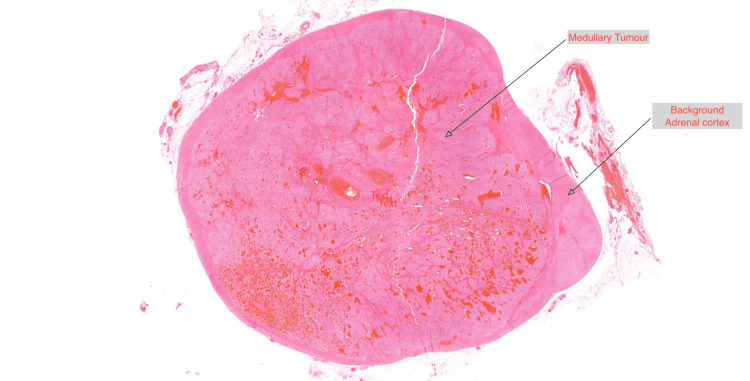
A cross-section of the adrenal gland showing a well-circumscribed medullary tumour with a rim of normal adrenal cortical tissue

**Figure 6:**
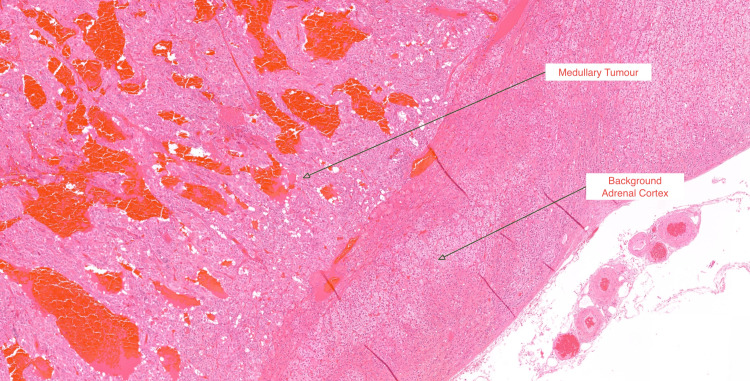
A medium-power view of the border between the medullary tumor (showing prominent vasculature and larger eosinophilic cells) and the background cortex

**Figure 7:**
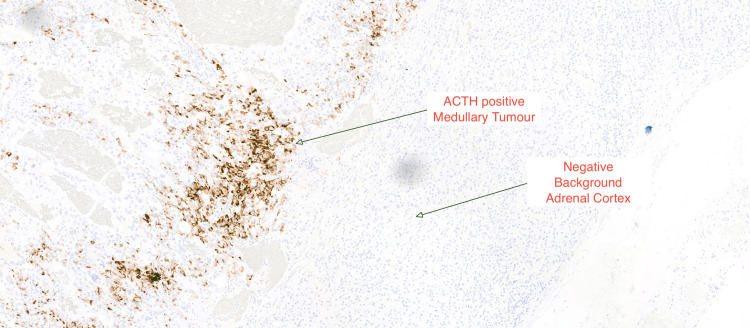
ACTH immunohistochemistry highlighting cells at the periphery of the medullary tumor ACTH: adrenocorticotropic hormone

The histopathology report revealed the presence of pheochromocytoma with low-risk histological features with patchy immunostaining for ACTH. The pheochromocytoma of the adrenal gland scaled score (PASS) was three (Figures 5, 6, 7) [6]. The gene panel (FH, MAX, MEN1, SDHA, SDHAF2, SDHB, SDHC, SDHD, TMEM127, VHL, and RET genes) analysis by next-generation sequencing did not identify any pathogenic variants associated with inherited pheochromocytoma or paraganglioma. At nine months post-surgery, her catecholamine profile remains within the normal range, and a repeat short Synacthen test remains suboptimal. Further follow-up will include an MRI of the sympathetic chain. 

## Discussion

CS secondary to ectopic production of ACTH by pheochromocytoma is extremely rare, occurring in approximately 100 patients reported in the literature [3]. About 80% of CS cases are caused by excess ACTH hormone, while the remaining 20% are due to abnormalities in the adrenal glands, usually an adrenal adenoma/carcinoma. Among the ACTH-related cases, most (80%-90%) are caused by a pituitary tumor, known as CS. In other cases, ACTH comes from outside the pituitary, called ectopic ACTH syndrome (EAS). The most prevalent tumors associated with ectopic ACTH secretion are small cell lung carcinomas (SCLC) (3.3% to 50%), bronchial carcinoid (5% to 40%), islet cell tumor of the pancreas (7.5% to 25%), thymic carcinoid (5% to 42%), and pheochromocytoma (2.5% to 25%) [7,8]. 

This diagnosis demands a high level of clinical suspicion and should be considered in the presence of severe metabolic derangement overlapping with CS's physical features. Because our patient had dual secretion of catecholamines and ACTH, her symptomatology was atypical. These tumors can be clinically challenging as the initial presentation may differ from a classical pheochromocytoma, which presents with headache, hypertension, diaphoresis, and tachycardia. These symptoms are often obscured by the rapid onset of hypercortisolism (hirsutism, proximal myopathy, and skin hyperpigmentation), which was one of the diagnostic clues in these patients. Consistent with our patient’s presentation, ectopic ACTH secretion can lead to profound metabolic disturbances such as hypokalemia, hyperglycemia, and hypertension [9]. 

Our patient presented with a three-week history of tremors and some features of CS, along with hypertension and new-onset diabetes. She was first referred to the diabetes team for management. The biochemical results of hypokalemia and metabolic alkalosis raised the suspicion of CS, which was then confirmed by significantly high cortisol levels. Initial work-up revealed ACTH-dependent hypercortisolism. Diagnostic clarity was further complicated by the CT of the abdomen and pelvis (performed to rule out a structural pancreatic cause for sudden hyperglycaemia), which showed a left solid heterogeneous adrenal nodule measuring 3.4 × 2.8 cm. The lesion was consistent with a pheochromocytoma, a diagnosis later supported by markedly elevated serum and urine metanephrines (Figure 2). MRI pituitary showed a normal pituitary gland and fossa. The whole body FDG PET/CT scan showed that the left adrenal nodule is intensely FDG avid, and there was diffuse increased uptake within both adrenal glands. An important clue was the lack of contralateral adrenal atrophy, which occurs because both adrenal glands are stimulated by ACTH. This finding points toward an ectopic source of ACTH rather than an adrenal adenoma or Cushing's disease.

Generally, a high-dose dexamethasone suppression test (HDDST) is performed to assist with differentiating between pituitary CS and ectopic CS. However, it is contraindicated in the setting of pheochromocytoma without appropriate alpha-adrenergic antagonism. Approximately 25 cases of phaeochromocytoma (PCC) crises associated with steroid administration have been reported in the literature [10]. Nonetheless, our patient underwent an uneventful HDDST, although it is important to note that exogenous glucocorticoids can precipitate severe psychiatric decompensation, particularly in individuals with underlying psychiatric disorders, as was the case for our patient. 

As the duration of hypercortisolism appears to be the most relevant determinant of morbidity and premature mortality, it also significantly influences symptom resolution and patient recovery, especially in terms of psychiatric outcomes [11]. The interval from symptom onset to diagnosis in CS varies by etiology, with ectopic sources associated with the shortest diagnostic delay (approximately 14 months), whereas pituitary-derived Cushing’s disease demonstrates the longest delay, averaging 38 to 45 months [3]. In contrast, the time from symptom onset to diagnosis in our patient was much shorter, at approximately seven weeks.

## Conclusions

ACTH-secreting pheochromocytomas are extremely rare causes of CS. This diagnosis should be considered in the presence of severe metabolic changes, particularly when the disease has a relatively rapid course and overlapping physical features of CS. It should be suspected in patients with ACTH-dependent CS, especially when imaging reveals an adrenal mass and biochemical tests suggest an ectopic source. Early diagnosis is crucial to reduce the risk of complications and mortality, as ACTH-producing pheochromocytomas can potentially be cured through surgery.
